# Exploring the Impact of Personalized Physical Therapy on a Patient With Motor Neuron Disorder: A Case Study

**DOI:** 10.7759/cureus.68373

**Published:** 2024-09-01

**Authors:** Saurabh N Puri, Raghumahanti Raghuveer, Shrushti Jachak, Priya Tikhile

**Affiliations:** 1 Department of Neuro-Physiotherapy, Ravi Nair Physiotherapy College, Datta Meghe Institute of Higher Education and Research (Deemed to be University), Wardha, IND; 2 Department of Musculoskeletal Physiotherapy, Ravi Nair Physiotherapy College, Datta Meghe Institute of Higher Education and Research (Deemed to be University), Wardha, IND

**Keywords:** amyotrophic lateral sclerosis, progressive bulbar palsy, rehabilitation strategies, physiotherapy rehabilitation, bulbar mnd, motor neuron disease

## Abstract

This case study examines the effect of a tailor-made physiotherapy regimen on an 85-year-old male patient who was suffering from bulbar motor neuron disease (MND) and had a history of stroke and COVID-19. The physiotherapy plan was designed to strategically address the patient's respiratory issues, generalized weakness affecting limb muscles, and speech and swallowing difficulties. Frequent evaluations made it possible to adjust the treatment plan, emphasizing a holistic strategy to improve the patient's overall quality of life. Improvements in scores on multiple functional scales and manual muscle testing were shown by outcome measures and follow-up evaluations. This case emphasizes how important customized physiotherapy is for maximizing functional outcomes and enhancing the quality of life for patients dealing with the complicated conditions of bulbar MND.

## Introduction

A collection of long-term, inherited, sporadic neurological conditions known as motor neuron disease (MND) is distinguished by the progressive degeneration of motor neurons. These may impact the motor neurons in the upper or lower limbs. The age at onset and the part of the central nervous system affected determine the prognosis of MND [1]. According to theory, MND is a progressive neurological condition that manifests as upper motor neuron signs (neurons that project from higher cortical centers to the brainstem and spinal cord) as well as lower motor neuron signs (anterior horn cells that project from the brainstem and the spinal cord to the muscle) [2]. Based on the site of origin and the degree of neurological involvement, there are four primary phenotypes of MND: primary lateral sclerosis, progressive bulbar palsy (PBP), amyotrophic lateral sclerosis (ALS), and progressive muscular atrophy [3]. With an incidence of two in 100,000 cases per year and a prevalence of five to seven per 100,000, MND is a relatively rare condition [4,5].

About 20% of patients have MND with bulbar onset. The first symptom is typically slurred speech due to poor tongue movement, accompanied by conspicuous tongue wasting and fasciculation. Dysphagia typically manifests later, after speech problems have become serious. Like other causes of pseudobulbar palsy, MND is frequently accompanied by emotional lability, which can show up as inappropriate crying or laughing. A pattern of onset where the respiratory muscles are affected first is the least common. Individuals may exhibit subtle clinical features such as frequent awakenings, unrefreshing sleep, hypersomnolence, and headaches in the morning, or they may exhibit dyspnea and orthopnea [6]. The general terms "ALS" and "MND" refer to a group of distinct phenotypes characterized by variable involvement of spinal and bulbar upper and lower motor neurons [7]. The degenerative and incapacitating neurological disease known as ALS causes the loss of motor neurons in the cortex, brainstem, and spinal cord [8]. Muscle weakness that impairs mobility and functionality, swallowing issues, respiratory muscle dysfunction, and ventilator insufficiency-related death are its hallmarks. Based on the initial onset of symptoms, two clinical classifications for ALS exist: spinal, which primarily affects the upper or lower limbs or both, and bulbar, which primarily affects the head and neck regions [9,10].

PBP, also known as bulbar phenotype, is characterized by bulbar onset accompanied by fasciculations, tongue wasting, dysphagia, and no involvement of the peripheral spinal cord during the first six months following the onset of symptoms [7]. Early in the course of the disease, progressive muscle weakness asymmetric in the upper and lower limbs can occur, reducing functional performance and adversely affecting quality of life [11]. While there are significant individual variations in this progression, it typically starts with the involvement of limbs and progresses to other body regions. Fatigue, which is defined as a generalized sense of exhaustion and difficulty achieving maximal muscle contraction, is another complaint made by people with ALS [12]. Most ALS patients are open to engaging in stretching and range-of-motion exercises. To accomplish therapeutic goals, low-to-moderate load resistance exercises for the unaffected muscles and submaximal aerobic activity can be useful.

## Case presentation

Patient information

The 85-year-old male patient was admitted to the hospital after having a stroke in April 2016. In 2020, he had COVID-19 and was hospitalized again. Since then, he has been dealing with hypertension and has been diagnosed with bulbar MND for the past 1.5 years. In October 2023, he was admitted to the neurology department at Acharya Vinoba Bhave Rural Hospital (AVBRH) due to difficulty in swallowing solid food and some discomfort when drinking liquids. After multiple investigations, physiotherapy was recommended for further treatment.

Clinical findings

**Figure 1:**
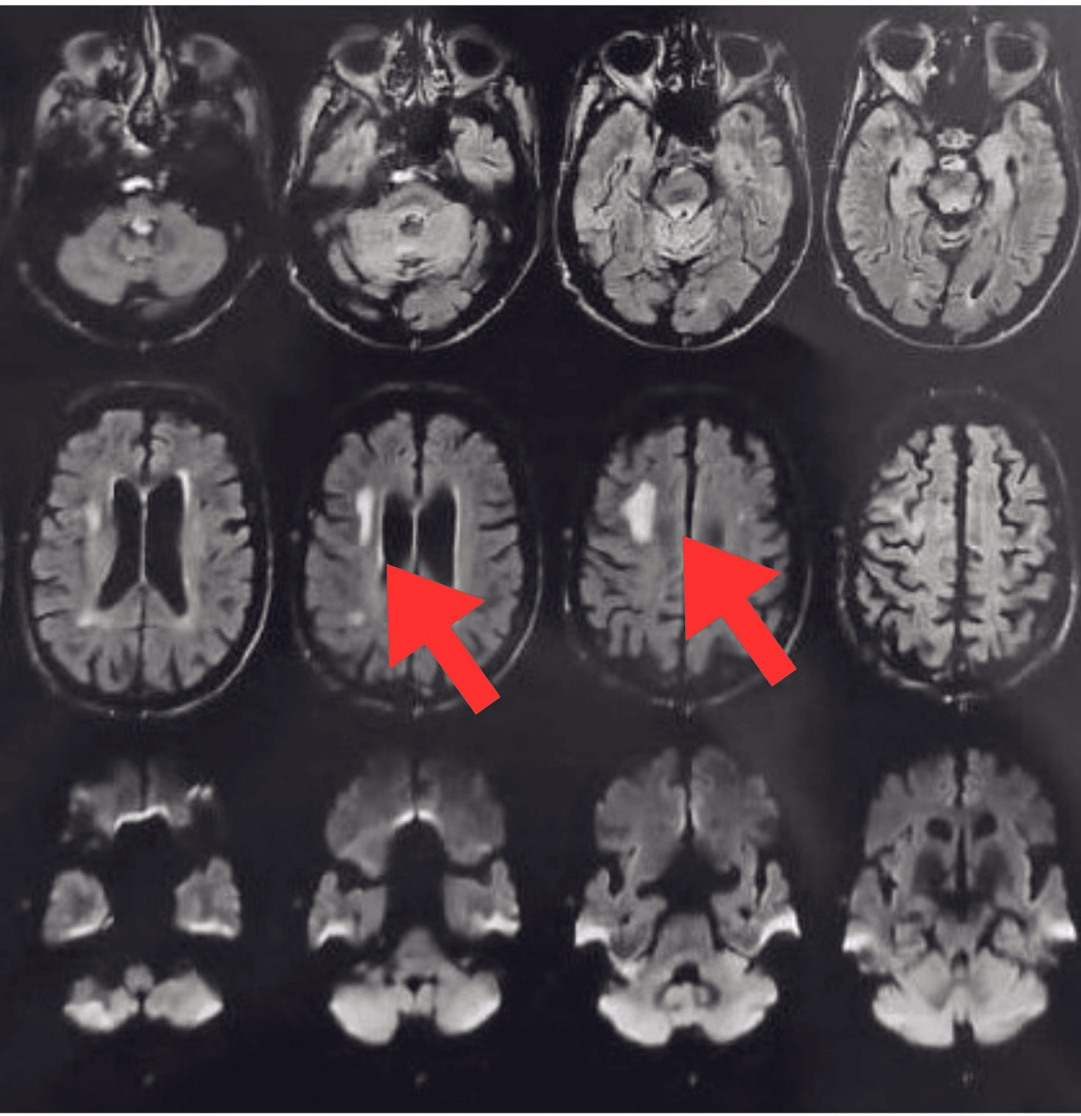
MRI shows ischemic changes

**Figure 2:**
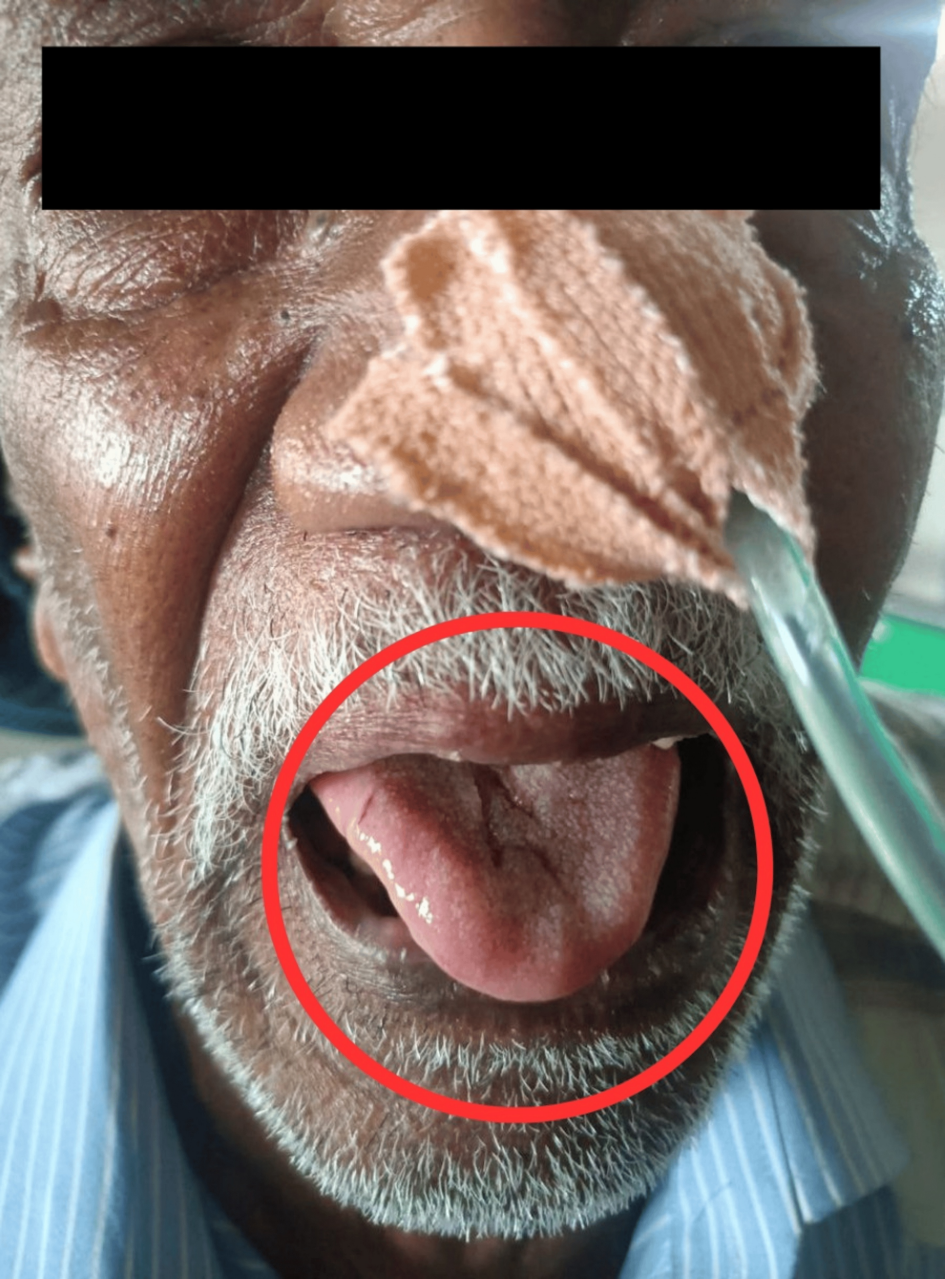
Deviation of tongue

The patient was aware and well-oriented, with a mesomorphic build. It is difficult to make facial expressions, smile, or tightly close eyes due to weak facial muscles. He showed intact sensations in both his upper and lower extremities. On the Medical Research Council Scale for Muscle Strength, there is a decrease in upper and lower limb muscular strength, which may indicate weakness. The investigations, including an MRI of the brain, show hyperintensities in the right high frontal region, centrum semiovale, and corona radiata, as well as age-related atrophy with small vessel ischemic diseases, as shown in Figure 1 with red arrow. X-ray and high-resolution CT (HRCT) scan of the thorax for COVID-19, which was done in 2020, show defined multiple patchy ground glass opacities with consolidation in both lungs with interlobar septal thickening and fibrotic bands. Electromyography (EMG) and nerve conduction velocity (NCV) tests show active and chronic partial denervation in the tongue, as shown in Figure 2, chronic partial denervation in both first dorsal interosseous, left tibialis anterior, and right medial gastrocnemius muscles, absent F response in both peroneal nerves, and absent sensory response in both sural nerves.

Physiotherapy interventions

Physiotherapy interventions for MNDs target respiratory difficulties, muscle weakness, and mobility impairments. Therapists concentrate on creating individualized exercise regimens that maximize joint flexibility, muscle strength, and coordination. In respiratory care, physiotherapists implement techniques to enhance lung function and guide respiratory exercises. To enhance general well-being and day-to-day functioning, the holistic approach consists of lifestyle modifications, patient education on adaptive techniques, and emotional support. When it comes to improving functional outcomes and quality of life for people with MNDs, physiotherapy is essential. Table 1 shows the complete rehabilitation protocol.

**Table 1:** Rehabilitation protocol

Component affected	Area affected	Cause	Goal	Physiotherapy treatment
Speech and swallowing	Bulbar region (speech and swallowing muscles)	Motor neuron degeneration	Maintain or improve speech and swallowing functions	Speech therapy, swallow exercises, adaptive strategies
Respiratory muscles	Diaphragm and intercostal muscles	Weakness due to motor neuron degeneration	Improve respiratory function	Breathing exercises, respiratory muscle training, assisted cough techniques
Facial muscles	Muscles controlling facial expressions	Motor neuron degeneration	Maintain or improve facial muscle strength	Facial exercises like cheek puffing, smile and frown exercise
Posture and mobility	Muscles throughout the body	Generalized weakness and loss of motor control	Improve or maintain mobility and posture	Range of motion exercises, strengthening exercises, gait training
Fatigue management	Overall body fatigue	Weakness and increased effort for movement	Reduce fatigue and enhance endurance	Energy conservation techniques, pacing, tailored exercise programs
Pain management	Muscles and joints	Altered biomechanics, muscle imbalances, and weakness	Alleviate pain and discomfort	Joint mobilization, soft tissue techniques, pain-relief strategies
Stretching components	Muscles throughout the body	Muscle tightness and contractures	Improve flexibility and prevent contractures	Passive stretching, active stretching, range of motion exercises
Strengthening components	Weak muscles due to motor neuron degeneration	Muscle atrophy and weakness	Maintain or improve muscle strength	Resistance exercises, functional strength training, isometric exercises
Emotional well-being	Psychological and emotional aspects	Progressive nature of the disease, impact on quality of life	Support emotional health and coping strategies	Counseling, support groups, mindfulness techniques, relaxation exercises

Follow-up and outcome measures

The outcome measure and the manual muscle testing (MMT) score improved, as shown in Tables 2-3.

**Table 2:** Scores from manual muscle testing before and after rehabilitation MMT: Manual Muscle Testing

MMT	Pre-rehabilitation		Post-rehabilitation	
	Right	Left	Right	Left
Shoulder	3+/5	3/5	4/5	4/5
Elbow	3+/5	3+/5	5/5	5/5
Wrist	3+/5	3+/5	4/5	4/5
Hip	3/5	3/5	4/5	4/5
Knee	3/5	3/5	4/5	4/5
Ankle	3+/5	3+/5	4/5	4/5

**Table 3:** Outcome measures and follow-up before and after treatment VAS: Visual Analog Scale; DOSS: Dysphagia Outcome and Severity Scale; FOIS: Functional Oral Intake Scale; HBS: House-Brackmann Score

Outcome measure	Pre-treatment scores	Post-treatment scores
VAS	7	3
DOSS	Level 2	Level 5
FOIS	3	5
HBS	Grade 4	Grade 3

## Discussion

The goal of rehabilitation methods for patients with bulbar MND is to keep them as functionally optimal as possible while avoiding complications brought on by immobilization and muscle atrophy [11]. The patient's multifaceted challenges posed by bulbar MND were strategically addressed in the physiotherapy plan. Targeted interventions in the speech and swallowing domain included using adaptive communication aids to improve effective interaction in addition to strengthening exercises for the muscles of the mouth and face. This all-encompassing strategy acknowledged the progressive nature of the dysphagia and dysarthria linked to bulbar MND to maintain current communication skills while also being flexible enough to accommodate evolving requirements [13,14].

Respiratory physiotherapy is needed to manage the respiratory compromise that comes with MND. Lung capacity-enhancing techniques were combined with coughing techniques that work, as they are essential for secretion clearance. Non-invasive ventilation techniques must also be used when needed to address the changing respiratory issues and preserve optimal respiratory function [15,16]. The physiotherapy plan identified the generalized weakness affecting limb muscles concerning overall mobility. Exercise programs specifically designed to target areas of weakness were put into place, and assistive technology was brought in to improve mobility and lower the risk of falls. By treating the physical effects of the illness, this strategy attempted to improve the overall quality of life in addition to preserving independence in daily activities [17-19].

A fundamental component that enabled dynamic modifications to the physiotherapy plan was the patient's condition, which was evaluated regularly. A cohesive approach that acknowledged the interplay of different symptoms and domains affected by bulbar MND was ensured through collaborative efforts with speech therapists and other healthcare professionals. This dynamic and comprehensive physiotherapy plan, created to maximize functional outcomes, demonstrated the dedication to improving the patient's overall health and quality of life despite the disease's progressive nature [20].

## Conclusions

For patients with bulbar MND, the objectives of physiotherapy interventions are to enhance overall health, minimize symptoms, and maximize function. It is essential to implement a multidisciplinary approach that promotes working together with respiratory specialists, speech therapists, and other medical specialists. This joint effort ensures that a comprehensive and individualized treatment plan designed for the unique challenges presented by bulbar MND will be developed. Physiotherapy, with its targeted and adaptable interventions, is essential to this comprehensive strategy. By addressing their specific needs, physiotherapy helps individuals with bulbar MND become more independent. This cooperative and patient-centered approach emphasizes the need for a strong healthcare team to deal with the complexities of this neurodegenerative condition.
